# Dynamic Response during PEM Fuel Cell Loading-up

**DOI:** 10.3390/ma2030734

**Published:** 2009-07-07

**Authors:** Pucheng Pei, Xing Yuan, Jun Gou, Pengcheng Li

**Affiliations:** State Key Laboratory of Automotive Safety and Energy, Tsinghua University, Beijing 100084, China; E-Mails: yuanx@mails.tsinghua.edu.cn (X.Y.); goujun_1997@163.com (J.G.); iamlpc@ae.buaa.edu.cn (P.L.)

**Keywords:** PEM fuel cell, loading-up, gas dynamic response, starvation

## Abstract

A study on the effects of controlling and operating parameters for a Proton Exchange Membrane (PEM) fuel cell on the dynamic phenomena during the loading-up process is presented. The effect of the four parameters of load-up amplitudes and rates, operating pressures and current levels on gas supply or even starvation in the flow field is analyzed based accordingly on the transient characteristics of current output and voltage. Experiments are carried out in a single fuel cell with an active area of 285 cm^2^. The results show that increasing the loading-up amplitude can inevitably increase the possibility of gas starvation in channels when a constant flow rate has been set for the cathode; With a higher operating pressure, the dynamic performance will be improved and gas starvations can be relieved. The transient gas supply in the flow channel during two loading-up mode has also been discussed. The experimental results will be helpful for optimizing the control and operation strategies for PEM fuel cells in vehicles.

## 1. Introduction

PEM fuel cells have gained much attention in recent years in their applications in transportation systems due to their characteristics of low operating temperature, quick start and high efficiency However, in order to reduce dependence on the battery, the fuel cell systems in vehicles often experience frequent load changes within a certain range, which may lead to local gas starvation in channels and potential damages to key components of the fuel cells [1]. Generally, gas starvations in the flow fields of fuel cells result from the sudden power output demands in the vehicles like start-ups or accelerations. These transient phenomena not only deteriorate the electric dynamic responses of the cells, but also contribute to the damage to the membrane electrode assembly (MEA) [2]. Therefore, a profound understanding of the effect of the transient gas supply or starvation on the load-up process is required both to improve dynamic performance and to prolong the lifetime of vehicular fuel cells. 

**NOMENCLATURE**Ucell voltage (V)Ttemperature (K)Iload current (A)
F
Faraday constant (-)△Ichange amplitude of electronic load current (A)△ttransient period during which the electronic load’s current changes (s)
$\lambda_{air}$
stoichiometric ratio in cathode of fuel cell (-)
${\dot{m}}_{O_{2}}$
mass flow rate of
$O_{2}$ usage (kg/s)ttime (s)
n
mole of the gas (mol)Pgas pressure (kPa)Rmolar gas constant (-)Vvolume of the gas (m^3^)



While many studies began recently to concentrate on the dynamic characteristics of fuel cells for transportation applications, little research has been conducted on the gas supply transients or starvation during the load-up process. The early dynamic model by Amphlett *et al*. [3], based on coupling the steady state electrochemical model and unsteady thermal model predicted the transient electric response of start-up, load-step and slow-down operating conditions. Most of the subsequent studies on dynamic processes [1,4,5,6,7,8], modeling [9,10,11], or testing [12,13,14,15,16], have focused on the transient behaviors of current-voltage polarization curves or local current distributions in flow fields [17,18]. However, few calculations or experiments have been conducted to investigate the transient phenomena of gas supply in the channels or the relationship of dynamic electric performance and local gas starvations. Though there have been many excellent CFD models that simulate the internal characteristics of the flow fields [19,20], most of them are limited to steady state flow conditions. Pathapati *et al*. [21] set up a transient mathematic model incorporating the dynamics of flow conditions in both anode and cathode sides and charge double layers to simulate the transients in flow fields and electric dynamic responses. However, only the average characteristic of dynamic flow conditions is presented and the effect of local distribution of reactants on the dynamic performance had not been identified. All these studies were carried out through modeling methods, while ones based on experimental data for the gas supply or starvation during dynamic processes are presently scarce. Kim *et al*. [22] presented experimental data and transient analysis on the dynamic response of PEM fuel cells and gas starvation during load changes. They examined the load change processes from the condition of gas excess to starvation, and also tested the effects of stoichiometric ratio on electric dynamic performance. The active area of the fuel cell in the experiments was only 25 cm^2^, which is far smaller than the actual active area of vehicular fuel cells. This work aims to study the transient characteristic of gas supply during the typical dynamic load-up process based on experimental approaches. Park *et al*. [23] presented a model for a 20-cell stack which was constructed to investigate start-up and transient behavior. Corbo *et al*. [24] showed that the best compromise is obtained between fuel cell system efficiency and dynamic response based on cell voltage regularity.

## 2. Experimental

This work is dedicated to studying the effects of the control and operating parameters of the load-up process on the gas supply and generating of gas starvation based on the dynamic behaviors of current outputs and voltage variations. Four parameters that should be of paramount importance for the load-up process, especially in vehicular fuel cell systems, have been studied. These parameters are the amplitude and rate of load changing, operating pressure and initial current density level. 

The single cell used in the experiments adopts serpentine channels in bipolar plates at both cathode and anode, which have an active area of 285 cm^2^. On the cathode side there are 16 channels that are 1.5 mm in width and 1.2 mm in depth, while there are seven channels that are 1 mm in width and 0.8 mm in depth on the anode side.

High purity hydrogen (99.99%) and industrial grade compressed air were used and the flow rates of two sides (anode and cathode) are controlled by an Alicat digital mass flow controller. The inlet gases are both humidified with two membrane humidifiers. Each humidifier consists of six membranes, which can control the temperature of the passing gases. An electronic load (ZSLV1502 H&H) is applied to consume the power output of the single cell and the load changing processes are also controlled by the signals from this equipment. All of the testing data are recorded by a LMS SCADAS recorder. The acquisition frequency can be up to 200 kHz. A testing PC is used to record various parameters during the experiments.

The control parameters of the load changing processes were tested in this article. As we know, fuel cell power systems always experience various load changing conditions. The rate of gas consumption in the flow field will increase immediately when there is a sudden increase of power output demand. When the power demand is changed with sufficiently large amplitude or quickly enough, reactant cannot be supplied in time and gas starvation occurs. In the experiments, different amplitudes and rates of current output variations were applied and the voltage responses investigated. 

Operating pressure affects the quantity of reactant gas stored in the channels, which will be consumed first during the load-up, so there will be more gas in local areas to supply for the reaction while the fuel cell is operating on a high level pressure and gas starvation is less likely occur. In the tests, three different operating pressures were applied to test the effect of pressure on this transient phenomenon.

During load changing process, the initial current value affects the initial reactant flux that can be a key factor in the gas quick supply and avoidance of gas starvation if the same stoichiometric ratios are given. In this part of tests, load changing processes with equal amplitudes of current variations and different initial current levels have been studied to demonstrate the effect of initial current value on gas starvation and the dynamic response of electric performance. 

## 3. Results and Discussion

### 3.1. Load-up amplitude effects

**Figure 1:**
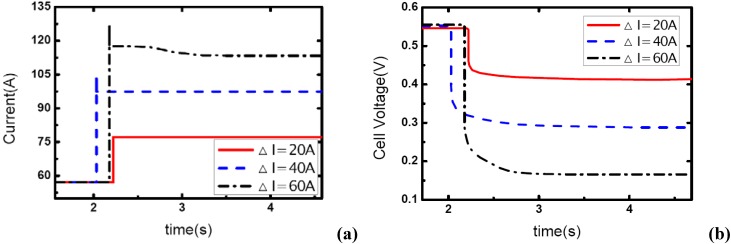
(a) Current change as three different step amplitudes while *λ_air_* ranges from 3 to 1.46, (b) corresponding dynamic voltage responses.

**Figure 2:**
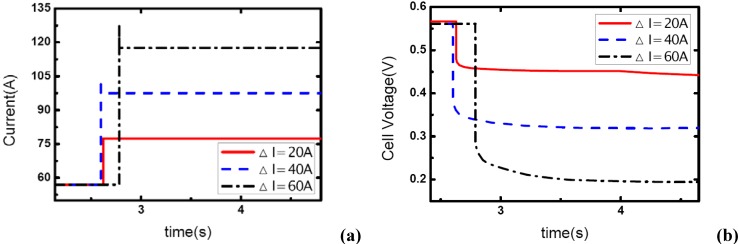
(a) Current change as three different step amplitudes while *λ_air_* ranges from 4 to 1.95, (b) corresponding dynamic voltage responses.

**Figure 3:**
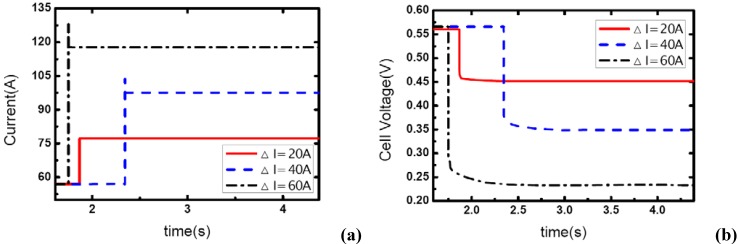
(a) Current change as three different step amplitudes while *λ_air_* ranges from 5 to 2.43, (b) corresponding dynamic voltage responses.

Figure 1, Figure 2 and Figure 3 show the step changes of current with three different amplitudes and the dynamic voltage responses for a single-cell PEM fuel cell. The temperature and operating pressure of the cell were 60 °C and 1.20 bar, respectively. The load-up processes are: 57A-77A, 57A-97A and 57A-117A, so the current variations ΔI are 20, 40 and 60 A, respectively. The stoichiometric ratios are constantly unity at the anode side due to the closing outlet of hydrogen flow field. For the cathode side, the ratios are set at 3, 4, and 5 prior to load changes for each process. 

In Figure 1, the load-up processes can be realized as expected while the current variations of the fuel cell are only 20 A and 40 A. For these two dynamic conditions, the current outputs can be step changed in a sufficiently short term – 10 ms, or even 1 ms. From the voltage curves, it can be seen that with the instant changes of current the voltages can also respond immediately due to the ohmic over-potential, then the curves decrease smoothly which should be understood as the concentration over-potential. It can also be seen that the concentration over-potential will take more time to reach the stable value when the amplitude of current variation increases from 20 A to 40 A. This is due to the fact that the rate of consumption of reactant gas in the channels depends on the current level, and the stored gas will be consumed more quickly when a higher current is drawn out, which leads to a greater concentration over-potential and more time is needed to reach the stable state when the inlet flow rate (here on the cathode side) has been fixed at a constant value.

More important is the dynamic behavior of current output and voltage response while the load-up amplitude increases up to 60 A. From Figure 1(a), the aiming current was attained for just a short time and then the output fell to a lower level. The failure of this load-up process resulted from the transient phenomenon of channel-gas starvation. Under this condition, the flow rate at the cathode side is set and the equivalent stoichiometric ratio is 3 prior to the dynamic process, therefore the ratio changes to 1.46 while the current output suddenly changes from 57 A to 117 A. Practically, the air ratio of 1.46 at the cathode can barely ensure a sufficient oxide supply to the reaction area and a homogeneous distribution of gas concentration in the flow field when a relatively high current is drawn out. In this case, local gas starvations may take place, which directly affect the current output. One point that should be mentioned here is that when the gas starvation appears and the current curve begins to decrease, the voltage curve would not fall anymore as under a lower current output level the voltage can be maintained stable.

Figure 2 and Figure 3 display the same load-up processes as Figure 1, except for the increase of the cathode stoichiometric ratio from 3 to 4 and 5. From these two figures, the trend explained above in which the over-potentials of the voltage curves and the time constant for the concentration over-potentials to achieve stabilization will both increase with the increase of load-up amplitude can be also clearly demonstrated. Compared with the condition of cathode stoichiometric ratio 3, all the load-up processes can be accomplished without any gas starvation when the stoichiometric ratio is raised to 4 or 5 before the load changes. Therefore, it can be concluded that for the load-up process of fuel cells, the most effective way to avoid gas starvation in the flow field should be by limiting the amplitudes of current output variations or ensuring sufficient reactant gas supply during this dynamic process.

### 3.2. Load-up rate effects

The effect of load-up rate on gas supply is studied in this part of experiment. Current output is changed from 57 A to 117 A. Δt is defined as the transient period during which the current changes from the initial value – 57A to the final value –117A, so Δt actually indicates the step changing rate of current output. Five different Δt values are used here to test the effect of the step changed rate of current on the dynamic behavior of electric performance and the transient gas supply. The operating pressure is set at 1.20 bar.

**Figure 4:**
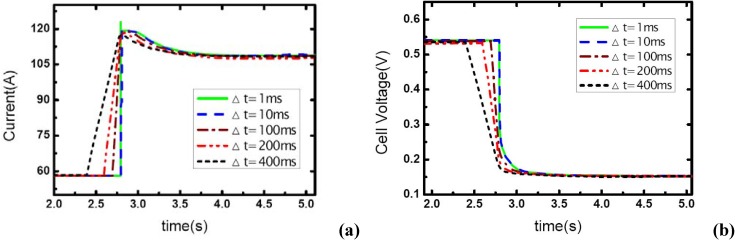
(a) Current change as five different step rates while *λ_air_* goes from 3 to 1.46, (b) corresponding dynamic responses of voltage.

Figure 4 shows the current step changes and corresponding voltage responses for the single-cell PEM fuel cell with the cathode stoichiometric ratios changing from 3.0 (cathode-rich) to 1.46 (cathode-starved). 

In Figure 4(a), the aiming current is maintained for only a short period after the step change, and then the current output falls down to a lower level. As described above, this transient phenomenon indicates the occurrence of gas starvation in channels. Prior to the load-up change process, reactant supply is sufficient at the cathode and extra gas is stored in the channels. The instant current output suddenly increases, the gas supply in the cathode is still sufficient for the transient reaction due to the stored gas in channels though it seems that *λ_air_* has changed from 3.0 to 1.46, from rich to starved. Therefore, the aiming current can be sustained immediately the load step changes. However, with the stored gas being consumed gradually, the gas supply becomes insufficient and the aiming current could not be maintained anymore. It also can be seen in the figure that the sustaining period decreases with the increase of Δt, as Table 1 shows. This phenomenon can be explained in terms of the stored gas in channels. Now that the quantity of stored gas in the flow field can be considered as a constant that can be given by ideal gas equation:

(1) PV=nRT

The stored gas will be also consumed during the rising edge of current before attaining the aiming value, so the sustaining period has to decrease as the rising time Δt increases. That means there will be less stored gas left in the channels to sustain the aiming current output while the rising edge of current takes more time and more stored gas has been consumed in the rising edge. The rate of consumption of reactant is given by:

(2) $${\dot{m}}_{O_{2}}=\frac{I}{4F}$$

where *F* is the Faraday constant 96,485.

Data in Table 1 also gives the percentage of stored gas consumed during the transient process (Δt and the sustaining period). From the comparison of the five cases, it can be concluded that the percentage of consumed gas are basically uniform despite some fluctuations, and the sustaining period of the higher level current is obviously affected by Δt or the load-on rate of fuel cell while the gas supply is insufficient and gas starvation follows.

**Figure 5:**
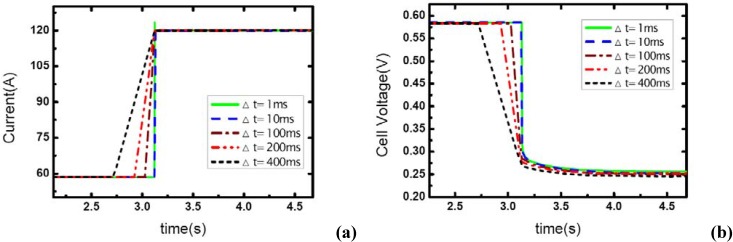
(a) Current change as five different step rates while *λ_air_* changes from 4 to 1.95, (b) corresponding dynamic responses of voltage.

**Figure 6:**
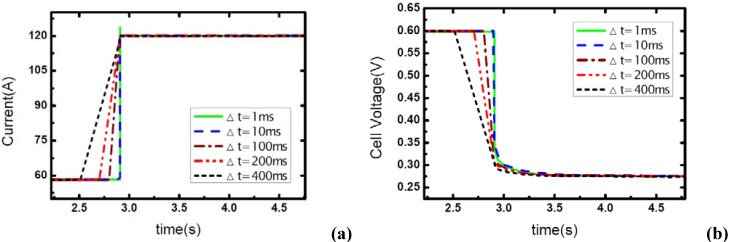
(a) Current change as five different step rates while *λ_air_* changes from 5 to 2.43, (b) corresponding dynamic responses of voltage.

In Figure 5 and Figure 6, the stoichiometric ratios of the cathode side prior to the dynamic process are raised to 4 and 5, respectively. So the *λ_air_* varies from 4 and 5 to 1.95 and 2.43, which means the gas supply at the higher level of current –117A changes to the state of nearly normal (1.95) and excess (2.43). From the figures, it can be seen that the load-up processes are accomplished successfully without gas starvations. Another important phenomenon is that concentration over-potentials of the voltage curves decreases as the load-up rate is slowed. This is due to the fact that the increase of Δt allows a full compensation of reactant gas during the rising edge of current and gas-consuming rate, which actually decreases the concentration over-potentials in a certain degree. As a conclusion, sufficient gas supply can ensure a no-starvation load-up process for fuel cells. Higher load-up rate will provide a longer sustaining period of the aiming current while gas starvation takes place, but lead to an increase of the concentration over-potentials of cell voltages while no starvations show up.

**Table 1:** Variations of Δt, sustaining time and consumed percentage of stored gas.

Case	Δt (ms)	Current Changing Rate (A/ms)	Sustaining Time (ms)	Consumed Percentage of Stored Gas (%)
1a	1	60	180	18.9
1b	10	6	160	17.4
1c	100	0.6	130	18.9
1d	200	0.3	50	16.6
1e	400	0.15	0	20

### 3.3. Pressure effects

**Figure 7:**
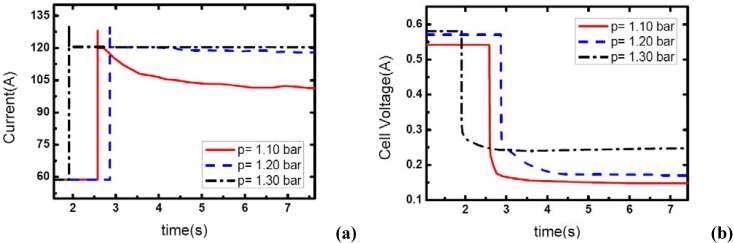
(a) Current change at three different operating pressures while *λ_air_* goes from 3 to 1.46, (b) corresponding dynamic responses of voltage.

**Figure 8:**
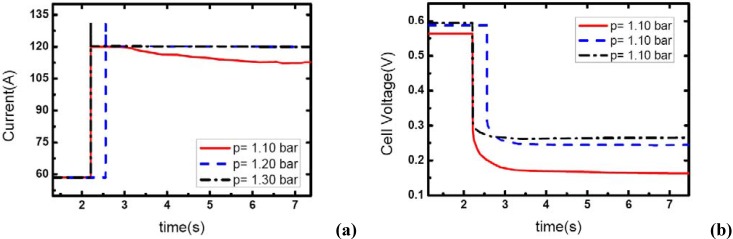
(a) Current change at three different operating pressures while *λ_air_* changes from 4 to 1.95, (b) corresponding dynamic responses of voltage.

**Figure 9:**
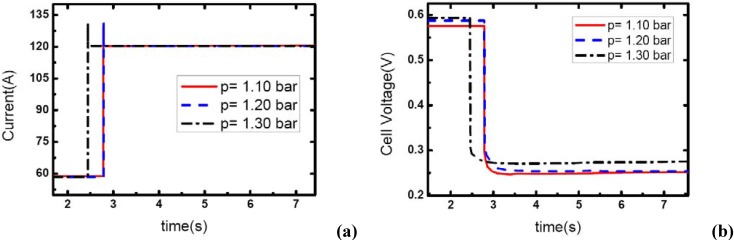
(a) Current change at three different operating pressures while *λ_air_* changes from 5 to 2.43, (b) corresponding dynamic responses of voltage.

Figure 7, Figure 8 and Figure 9 show the effect of operating pressure on the dynamic electric responses of the fuel cell and gas supply. The dynamic process applied in the experiment is the same as discussed above 57 A-117 A, and the cathode stoichiometric ratios before load changing are set at 3, 4 and 5. Three different operating pressures are applied: 1.10, 1.20 and 1.30 bar. 

In Figure 7, the cathode stoichiometric ratio changes from 3.0 (reactant rich) to 1.46 (reactant starved). Similarly, two of the current curves display the same behavior that indicates gas starvation as the ones studied in the above tests. However, the load-up process is obtained successfully as the operating pressure increases to 1.30 bar while in terms of the variation of *λ_air_*, gas starvation in the flow field should have taken place. This may be due to the quick compensation of gas supply and high-usage of gas in the reaction under relatively high operating pressure. As we know, increasing the operating pressure will make the fundamental reaction more active and lead to a fruitful utilization of inlet gas. Therefore, the condition of gas supply to the reaction areas can be improved while the operating pressure increases.

From Figure 7(b), the general effect of operating pressure on the electric performance can be inferred. With the increase of pressure, the cell voltage increases accordingly. This is due to the fact that higher operating pressure can lower both the activation over-potential and mass transport over-potential, generally are understood as a part of the concentration over-potential. 

Figure 8 and Figure 9 show the similar dynamic process with the cathode stoichiometric ratio rises to 4 and 5 prior to the load-up process, respectively. Compared with the cases shown in Figure 4, the gas starvation under the condition of *λ_air_*=3 and operating pressure 1.20 bar has disappeared here due to the increase of *λ_air_*. However, for the operating pressure of 1.10 bar, the gas supply during the dynamic process is still insufficient and gas starvation appears. With the cathode stoichiometric ratio increasing up to 5, gas starvations in the channels vanish completely and all the load-up processes can be attained successfully. Therefore, it can be concluded that operating pressure is a key factor to affect the generating of gas starvation during the load-up process. Higher operating pressure can relieve the gas starvation under an insufficient gas flow supply during load-up process. Increasing the operating pressure can compensate for the decrease of reactant stoichiometric ratio in avoiding gas starvations in the flow field, especially on the cathode side.

### 3.4. Initial current effects

**Figure 10:**
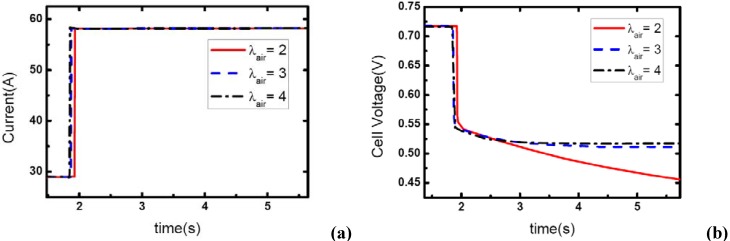
(a) Current change at three different stoichiometric ratios in cathode when the initial current is 28.5 A, (b) corresponding dynamic responses of voltage.

**Figure 11:**
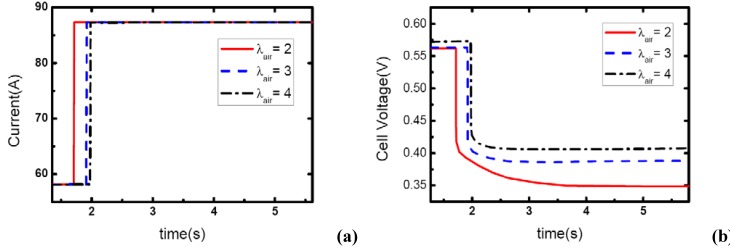
(a) Current change at three different stoichiometric ratios in cathode when the initial current is 57 A, (b) corresponding dynamic responses of voltage.

**Figure 12:**
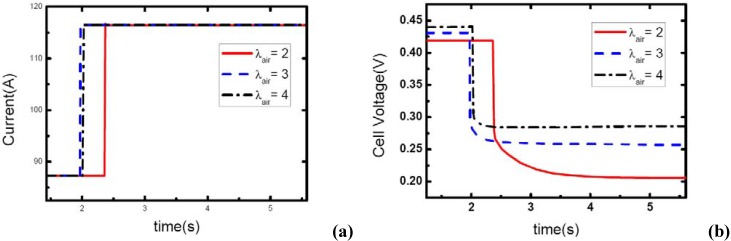
(a) Current change at three different stoichiometric ratios in cathode when the initial current is 88.5 A, (b) corresponding dynamic responses of voltage.

Figure 10, Figure 11 and Figure 12 show the step changes of current and dynamic responses of cell voltage during the load-up processes with the same amplitude of current variation and stoichiometric ratios on both sides, but different initial values of current output. The results display the effect of operating current level on the dynamic characteristic of electric performance of fuel cell during load-up processes on vehicles. In each figure, the effect of *λ_air_* on dynamic gas supply and electric performance can also be demonstrated.

In Figure 10(b), the voltage curves of the fuel cells are enhanced with the increase of *λ_air_*, which should be due to the increasing gas supply for the reaction. Specifically, the voltage curve can hardly be maintained after the current step changing while the cathode stoichiometric ratio changes from 2 to 1 during this process. This should be due to the fact that a serious gas starvation may have taken place at this moment; however, the high value of cell voltage ensures that the current output could be maintained for a certain period until the starvation is relieved by dynamic reactant supply from the inlet of the channels.

**Figure 13:**
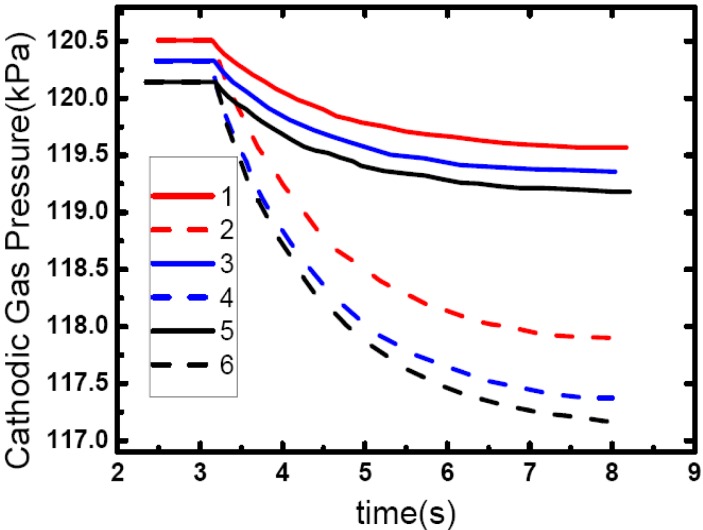
Cathode gas dynamic response while in direct loading-up. (1) Channel inlet when current is changed from 60 A to 82 A. (2) Mid-channel when current is changed from 60 A to 82 A. (3) Channel outlet when current is changed from 60 A to 82 A. (4) Channel inlet when current is changed from 60 A to 126 A. (5) Mid-channel when current is changed from 60 A to 126 A. (6) Channel outlet when current is changed from 60 A to 126 A.

As the same setting of cathode stoichiometric ratios (2, 3 and 4) prior to load-up and the same amplitude of current step change are applied, the cathode stoichiometric ratios after load changing will be improved with the increase of initial current. For example, during the load-up process: 28.5 A-57 A, *λ_air_* changes from (2, 3, and 4) to (1, 1.5, and 2) respectively; while for the process of 85.5 A – 114 A, *λ_air_* changes from (2, 3, and 4) to (1.5, 2.25, and 3) respectively. Compared with the condition shown in Figure 11, gas starvation has disappeared and load-up processes can be obtained smoothly as the cathode stoichiometric ratios increased, as shown in Figure 12 and Figure 13. The reason why some cases here with cathode stoichiometric ratios lower than 1.5 do not show the gas starvations like the case 1c in Table 1 may be due to the decreased load-up amplitude ΔI=28.5 A. It can also be seen from the voltage curves that concentration over-potentials decrease with the initial value of current in this transient process. This is mainly due to the increased level of cathode stoichiometric ratios and sufficient gas supply in the flow field during the load-up process. Therefore, while appropriate stoichiometric ratios are employed by the induction controlling system of fuel cell, improving the level of operating current will bring an adequate reactant supply, effectively avoid gas-starvation and smooth the load-up process.

### 3.5. Cathode gas dynamic response

**Figure 14:**
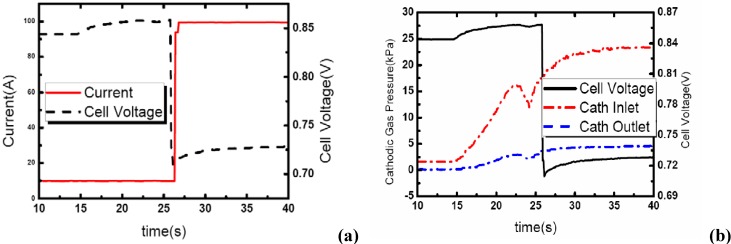
Dynamic response when in delayed loading-up mode for (a) current and voltage (b) cathode gas pressure.

As shown in Figure 13, cathode gas pressure is gradually going down while the PEM fuel cell loading-up. Also, Figure 14 shows that the greater the amplitude of the loading-up process is, the more the cathode gas pressure changes, and the pressure in the outlet of flow channel changes more than that in the inlet, which would make some cells undergo starvation while car overtaking, accelerating or climbing, and make cell performance degrade rapidly.

Another delayed loading-up mode was obtained in experiments on another PEM fuel for comparison. As shown in Figure 14(a), in this delayed loading-up mode, current changes later than voltage, while current and voltage changes almost the same time in direct loading-up mode shown in Figure 12. Also, in Figure 14(b), in this delayed loading-up mode, cathode gas pressure is gradually increasing, while it is going down in the direct loading-up mode shown in Figure 13, and pressure in the outlet changes less than in the inlet when the pressure in the outlet changes more than in the inlet in the direct loading-up mode shown in Figure 13.

The delayed loading-up mode could be adopted in automobile fuel cells to avoid starvation, and extend the lifetime of fuel cells. But, as flux suddenly increases in the inlet during PEM fuel cell loading-up, the membrane would dry out, which would also make cell performance degrade rapidly. Furthermore, this mode needs a powerful gas supply system to satisfy the fast dynamic response during PEM fuel cell loading-up.

## 4. Conclusions

A study of the effects of four controlling and operating parameters on dynamic gas supply during the load-up process for a PEM fuel cell is presented based on experimental data. The dynamic behaviors of current output and voltage responses are observed and transient gas supply, even starvation in the flow field is analyzed. The results indicate that increasing the amplitude of load-up would both improve concentration over-potential and the possibility of gas starvations in channels when a constant flow rate has been set for the cathode. A higher load-up rate provides a longer sustaining period of the aiming current as gas starvation takes place, but brings about an increase of the concentration over-potentials of cell voltages while no starvations appear. With a higher operating pressure, the dynamic performance could be improved and gas starvations are also relieved under an insufficient gas supply during the load-up process. The possibility of gas starvation and the concentration over-potential during the load-up process both decrease with the initial current level increasing while the same current step changing amplitude is applied and constant cathode stoichiometric ratios are fixed. All the rules of dynamic electric performance and gas supply during load-up process deduced from the experiments will be helpful for optimizing the controlling and operating strategies of PEM fuel cells on vehicles.

## References

[1] Kulikovsky A.A., Scharmann H., Wippermann K. (2004). Dynamics of fuel cell performance degradation. Electrochem. Commun..

[2] Jiang R., Chu D. (2001). Voltage-time behavior of a polymer electrolyte membrane fuel cell stack at constant current discharge. J. Power Sources.

[3] Amphlet J.C., Mann R.F., Peppley B.A., Roberge P.R., Rodrigues A. (1996). A model predicting transient responses of proton exchange membrane fuel cells. J. Power Sources.

[4] Kim S., Shimpalee S., Van Zee J.W. (2004). The effect of stoichiometry on dynamic behavior of a proton exchange membrane fuel cell (PEMFC) during load change. J. Power Sources.

[5] Yerramalla S., Davari A., Feliachi A., Bisis T. (2003). Modeling and simulation of the dynamic behavior of a polymer electrolyte membrane fuel cell. J. Power Sources.

[6] Wang Y., Wang C.Y. (2005). Transient analysis of polymer electrolyte fuel cells. Electrochim. Acta.

[7] Wu H., Lia X.G., Berg P. (2007). Numerical analysis of dynamic processes in fully humidified PEM fuel cells. Int. J. Hydrogen Energ..

[8] Weydahl H., Møller-Holst S., Hagen G., Børresen B. (2007). Transient response of a proton exchange membrane fuel cell. J. Power Sources.

[9] Haddad A., Bouyekhf R., El Moudni A. (2008). Dynamic modeling and water managementin proton exchange membrane fuel cell. Int. J. Hydrogen Energ..

[10] Hung Y.H., Lin P.H., Wu C.H., Hong C.W. (2008). Real-time dynamic modeling of hydrogen PEMFCs. J. Franklin Inst.-Eng. Appl. Math..

[11] Shan Y.Y., Choe S.-Y. (2005). A high dynamic PEM fuel cell model with temperature effects. J. Power Sources.

[12] Lee Y.T., Kim B.S., Kim Y.C. (2009). Effects of self-humidification on the dynamic behavior of polymer electrolyte fuel cells. Int. J. Hydrogen Energ..

[13] Qu S.G., Li X.J., Hou M., Shao Z.G., Yi B.L. (2008). The effect of air stoichiometry change on the dynamic behavior of a proton exchange membrane fuel cell. J. Power Sources.

[14] Shen Q., Hou M., Yan X.Q., Liang D., Zang Z.M., Hao L.X., Shao Z.G., Hou Z.J., Ming P.W., Yi B.L. (2008). The voltage characteristics of proton exchange membrane fuel cell (PEMFC) under steady and transient states. J. Power Sources.

[15] Cho J.H., Kim H.-S., Min K.D. (2008). Transient response of a unit proton-exchange membrane fuel cell under various operating conditions. J. Power Sources.

[16] Yan Q.G., Toghiani H., Causey H. (2006). Steady state and dynamic performance of proton exchange membrane fuel cells (PEMFCs) under various operating conditions and load changes. J. Power Sources.

[17] Mench M.M., Dong Q.L., Wang C.Y. (2003). In situ water distribution measurements in a polymer electrolyte fuel cell. J. Electrochem. Soc..

[18] Stumper J., Campbell S.A., Wilkinson D.P., Johnson M.C., Davis M. (1998). In-situ methods for the determination of current distributions in PEM fuel cells. Electrochimica Acta.

[19] Nguyen P.T., Berning T., Djilali N. (2004). Computational model of a PEM fuel cell with serpentine gas flow channels. J. Power Sources.

[20] Maharudrayya S., Jayanti S., Deshpanda A.P. (2004). Pressure losses in laminar flow through serpentine channels in fuel cell stacks. J. Power Sources.

[21] Pathapati P.R., Xue X., Tang J. (2005). A new dynamic model for predicting transient phenomena in a PEM fuel cell system. Renewable Energy.

[22] Kim S., Shimpalee S., Van Zee J.W. (2004). The effect of stoichiometry on dynamic behavior of a proton exchange membrane fuel cell (PEMFC) during load change. J. Power Sources.

[23] Park S.-K., Choe S.-Y. (2008). Dynamic modeling and analysis of a 20-cell PEM fuel cell stack considering temperature and two-phase effects. J. Power Sources.

[24] Corbo P., Migliardini F., Veneri O. (2008). Experimental analysis of a 20 kW PEM fuel cell system in dynamic conditions representative of automotive applications. Energ. Conv. Manage..

